# Biological Potential of *Hypericum* L. Sect. *Drosocarpium* Species

**DOI:** 10.3390/life15081332

**Published:** 2025-08-21

**Authors:** Nebojša Kladar, Branislava Srđenović Čonić, Goran Anačkov, Maja Hitl, Bojana Bokić, Boris Radak, Milica Rat

**Affiliations:** 1 Department of Pharmacy, Faculty of Medicine, University of Novi Sad, Hajduk Veljkova 3, 21000 Novi Sad, Serbia; branislava.srdjenovic-conic@mf.uns.ac.rs (B.S.Č.); maja.bekut@mf.uns.ac.rs (M.H.); 2 Center for Medical and Pharmaceutical Investigations and Quality Control, Faculty of Medicine, University of Novi Sad, Hajduk Veljkova 3, 21000 Novi Sad, Serbia; 3 Department of Biology and Ecology, Faculty of Sciences, University of Novi Sad, Trg Dositeja Obradovica 2, 21000 Novi Sad, Serbia; goran.anackov@dbe.uns.ac.rs (G.A.); bojana.bokic@dbe.uns.ac.rs (B.B.); boris.radak@dbe.uns.ac.rs (B.R.); milica.rat@dbe.uns.ac.rs (M.R.)

**Keywords:** antioxidant, acetylcholinesterase, monoamine oxidase, α-amylase, α-glucosidase

## Abstract

The limited data on biological potential of the genus *Hypericum* sect. *Drosocarpium* species initiated the current research aimed at the chemical characterization of samples of six selected taxa (*H. barbatum*, *H. montbretii*, *H. richerii* subsp. *grisebachii*, *H. rochelii*, *H. rumeliacum*, and *H. spruneri*) and the evaluation of their biological potential (antioxidant and antihyperglycaemic potential, acetylcholinesterase and monoamine oxidases inhibition). The obtained results suggest greater abundance of biologically active compounds, hypericin (*H. rochelii*, *H. barbatum*, and *H. richerii* subsp. *grisebachii*), amentoflavone (*H. richerii* subsp. *grisebachii*), quercetin and rutin (*H. richerii* subsp. *grisebachii*), and chlorogenic acid (*H. richerii* subsp. *grisebachii*, *H. barbatum*, *H. rumeliacum*), when compared to *H. perforatum*. Also, the scavenging potential of DPPH (median RSC_50_ = 3.34 µg/mL), NO (median RSC_50_ = 26.47 µg/mL) and OH radicals (median RSC_50_ = 76.87 µg/mL) of evaluated species was higher, or at least comparable to *H. perforatum*, while the same trend was noticed in the case of anti-MAO-A (median IC_50_ = 19.41 µg/mL) and antihyperglycaemic potential (inhibition of α-amylase and α-glucosidase (median IC_50_ = 29.47 µg/mL)). The study results highlight sect. *Drosocarpium* species as a valuable source of biologically active secondary metabolites and suggest a wide spectrum of possible applications in the food and medicine industries.

## 1. Introduction

The *Hypericum* L. genus is one of the most important medicinal plants genera. Although represented by more than 500 widely geographically distributed species, only St. John’s wort (*H. perforatum*, Hypericaceae) has been extensively studied [1,2]. This increased research popularity has resulted in numerous evidence-based data describing St. John’s wort preparations’ biological, pharmacological, and clinical potential. Specifically, these preparations have demonstrated antioxidant, anti-inflammatory, anti-cancer, antibacterial, antiviral, anticholinesterase, analgesic, antidepressant, hepatoprotective, and antihyperglycemic effects [3]. St. John’s wort water–alcoholic extract is clinically effective in the treatment of mild to moderate forms of depression, while oil macerate is being predominantly utilized by traditional medicine, applied externally for the treatment of wounds, bruises, eczema, or internally for relieving gastrointestinal ulcerations, biliary disorders, diabetes, migraines, or headaches [2]. Moreover, the European Medicines Agency states the traditional application of St. John’s wort preparations in the treatment of insomnia and temporary mental exhaustion. The presented medicinal potential of St. John’s wort is a result of several classes of secondary metabolites, such as naphthodianthrones, phloroglucinols, phenolic acids, flavonoids and their glycosides, biflavonoids, and xanthones [4]. However, the aforementioned secondary metabolites are not only characteristic of *H. perforatum* but are also widely distributed among other species of the genus. This strongly supports the latest reports on applying various *Hypericum* species (other than St. John’s wort) in the traditional medicine of Europe, Africa, Asia, and America as analgesics, antidepressants, diuretics, astringents, and antipyretics [1,4,5]. Moreover, phytochemical studies in the genus *Hypericum* have so far led to the isolation of more than 400 compounds with confirmed in vitro and/or in vivo biological potential [2], allowing us to highlight this genus as a significant resource for future studies in the area of natural products with medicinal properties. The antioxidant potential of secondary metabolites present in *Hypericum* species is of interest for potential utilization in the food and cosmetics industry and in terms of medicinal application. Highly specific biological activities demonstrated for *Hypericum* species, such as inhibition of monoamine oxidases (MAOs) and α-glucosidase, require particular attention, since they are being followed by strong antioxidant activity. Briefly, the inhibition of previously mentioned enzymes is a recognized pharmacological approach in treating neurodegenerative diseases and diabetes mellitus type 2, respectively, in which the increased oxidative stress represents a significant factor influencing disease management.

The most recent systematics of the genus *Hypericum* classifies all species in 36 taxonomic sections [1]. Resemblance in qualitative chemical profiles of St. John’s wort and other *Hypericum* species can be expected if these species belong to some of the phylogenetically younger sections [6]. Moreover, of particular importance are the *Hypericum* species which accumulate higher amounts of biologically active secondary metabolites when compared to *H. perforatum*, since they could be expected to exhibit more notable biological activity or to represent a valuable raw material for the extraction of specific compounds [7]. A prominent example complying with the criteria above is the representatives of the *Drosocarpium* section. A total of 11 species geographically distributed in the area between the northwestern coast of Africa and the Black Sea regions of Turkey and Georgia are characteristic for dense coverage of leaves and flowers by dark glands, known for accumulating naphthodianthrones (hypericin and its derivatives) [8]. Hypericin has been recognized as an important biologically active secondary metabolite which increases extracellular glutamate and acetylcholine levels, thus affecting stress induced conditions, but also exhibits anti-cancer activity [2]. Furthermore, the available data on phytochemical profiling of some species from the *Drosocarpium* section also suggest the presence of other compounds (hyperforin, flavonoids, phenolic acids) of interest regarding the *Hypericum* genus [9]. However, to our knowledge, most of the *Drosocaproium* species have not been studied or have only been subjected to different preliminary chemical characterization experiments [7], while at the same time there are reports of their application in traditional medicine (i.e., *H. richerii* subsp. *grisebachii* and *H. barbatum* in folk medicine of Montenegro and Italy) as an effective substitute of St. John’s wort [5,10,11].

Current research aims to perform chemical characterization of six *Hypericum* species from the *Drosocarpium* section (*H. barbatum* Jacq. 1775, *H. montbretii* Spach 1836, *H. richerii* Vill. 1779 subsp. *grisebachii* (Boiss.) Nyman 1878, *H. rochelii* Griseb. et Schenk 1852, *H. rumeliacum* Boiss. 1849, and *H. spruneri* Boiss. 1849) represented by a total of 44 samples collected on the territory of the central part of the Balkan Peninsula, as well as perform screening of their biological potential (antioxidant, antihyperglycemic, anticholinergic, inhibition of monoaminoxidase). These species are, according to available reports, expected to display a significant accumulation of secondary metabolites characteristic for *Hypericum* genus and, consequently, notable biological potential, but so far have not been extensively studied.

## 2. Materials and Methods

### 2.1. Chemicals and Reagents

2,2-diphenyl-1-picrylhydrazyl radical, acetylthiocholine iodide, iron(II)-sulfate heptahydrate, iron(III)-chloride hexahydrate, N-(1-naphthyl)-ethylenediamine dihydrochloride, and sulfanilamide were purchased from Alfa Aesar (Haverhill, MA, USA); Folin–Ciocalteu (FC) reagent was obtained from Merck (Darmstadt, Germany); acetylcholinesterase solution was obtained from Roche (Basel, Switzerland); 2-thiobarbituric acid, ethanol, ethylenediaminetetraacetic acid disodium salt dihydrate, formic acid, hydrochloric acid, hydrogen peroxide, methanol, potassium dihydrogen phosphate, potassium hydrogen phosphate, sodium carbonate, sodium nitroprusside, and trichloroacetic acid were purchased from POCH (Gliwice, Poland); 2,4,6-tris(2-pyridyl)-S-triazine, 2-deoxy-D-ribose, acarbose, aluminum chloride, ascorbic acid, butylated hydroxytoluene, caffeic acid, chlorogenic acid, epicatechin, ferulic acid, galantamine, gallic acid, glutathione (reduced), moclobemide, p-hydroxybenzoic acid, p-nitrophenyl-α-D-glucopyranoside, propyl gallate, quercetin, quercetin dihydrate, rutin, selegiline, Starch azure, α-amylase, α-glucosidase, MAO-A, and MAO-B were obtained from Sigma Aldrich (St. Louis, MO, USA); and amentoflavone, hypericin, and hyperforin were purchased from Extrasynthese (Genay Cedex, France).

### 2.2. Plant Material and Preparation of Extracts

Six species of the genus *Hypericum* sect. *Drosocarpium* (*H. barbatum*, *H. montbretii*, *H. richerii* subsp. *grisebachii*, *H. rochelii*, *H. rumeliacum*, and *H. spruneri*), represented by a total of 44 samples, were collected on the territory of the central part of the Balkan Peninsula. The voucher specimens of collected samples (Table S3) were taxonomically identified and deposited by the BUNS Herbarium (Herbarium of the Department of Biology and Ecology, Faculty of Natural Sciences and Mathematics, University of Novi Sad).

The habitats of *H. barbatum* samples were highly variable. Samples b1–b3 and b9–b11 were growing on silicate bedrock, with reddish, acidic mountain soil at approximately 1000 m above sea level (a.s.l.), in sparse *Pinus nigra* forest, while the herbaceous layer was dominated by overgrown tussock-forming grasses, within submontane to montane vegetation zones. Samples b4–b6 and b8 were collected at similar altitude, but on open steppic grasslands on limestone bedrock, within the submontane to montane vegetation zones. The sample b7 was characterized by the habitat of the deciduous mixed forest, along the road, at approximately 830 m a.s.l. *H. montbretii* samples were collected at approximately 500 m a.s.l. in edge habitats formed at the interface of deciduous mixed forests and serpentine roads. *H. richerii* subsp. *grisebachii* habitats included open steppic grasslands developed on limestone bedrock, occurring at elevations above 1500 m a.s.l., within the submontane to montane vegetation zones. Samples of *H. rumeliacum* were collected on dry, open grasslands on serpentine bedrock, occurring at the foothills of mountains, in valleys, or canyon bottoms, while *H. spruneri* samples were characterized by sunny, rocky roadcut habitats on calcareous bedrock, with sparse shrub and ruderal vegetation. *H. rochelii* samples were collected on open montane–subalpine grasslands on limestone bedrock, occurring above the upper forest limit (approximately 1200–1500 m a.s.l.) on steep slopes (ro1 and ro3 samples), or on open steppic grasslands on calcareous bedrock (ro2, ro4, ro5 samples). Plants’ upper aerial parts collected at full flowering stage were dried, ground (sieve 355 μm), and, according to European Pharmacopeia, macerated (drug: solvent = 1:5) at 25 °C for 72 h with ethanol (70%, *w*/*w*) [12]. The obtained extracts were evaporated under vacuum (Rotavapor R-100, BÜCHI, Flawil, Switzerland), and the extraction yield was quantified. The resulting dried extracts were dissolved in distilled water at a concentration of 10% (*w*/*w*) and used for evaluation of biological potential. On the other hand, chemical characterization of prepared extracts was performed after dissolving them in methanol (50%, *w*/*w*).

### 2.3. Phytochemical Analysis of Plant Extracts

The content of total phenolics (TPC) and flavonoids (TFC) in the studied extracts was quantified according to spectrophotometric assays published by Bozin et al. [13], using Folin–Ciocalteu reagent for total phenolics, and aluminum chloride as a complexing reagent for flavonoids determination. The results were expressed in mg of gallic acid equivalents (GAE) per g of dry extract (mg GAE/g d. e.) and mg of quercetin equivalents (QE) per g of dry extract (mg QE/g d. e.), respectively.

Detailed quantitative chemical characterization of the analyzed extracts was performed using previously reported HPLC–DAD (High-performance liquid chromatography coupled to diode-array detection) analytical methods. The first method [14] was used for the quantification of hypericin, hyperforin, and amentoflavone, while the second [15], slightly modified according to the parameters described in the paper published by Kladar, Božin, Bijelić, Bogavac, Karaman, Srđenović Čonić, Rat, and Anačkov [3] was used for the determination of rutin, quercetin, epicatechin, and selected phenolic acids, such as gallic, chlorogenic, caffeic, ferulic, and *p*-hydroxybenzoic acid. Both analyses were performed on an Agilent HP 1100 instrument (Agilent, Waldbronn, Germany) using a Zorbax CB-C18 column (4.6 × 150 mm, 5 µm particle size). The results were expressed as µg/g of dry herbal material (d. h.).

### 2.4. Biological Potential Evaluation

#### 2.4.1. Antioxidant Potential Evaluation

The ability of extracts to scavenge free radicals was investigated using previously described in vitro assays based on scavenging DPPH, NO, and OH radicals [3,16]. DPPH assay relies on the decrease in the absorbance at 515 nm, which occurs due to DPPH radical (c(DPPH^•^) = 25 μM) reduction by added herbal extracts. Nitroso radicals (NO^•^) are formed in the test system by the addition of sodium nitroprusside (c(NO^•^) = 3.5 mM) and increasing concentrations of analyzed extracts are added. The free NO^•^, which was not reduced by the addition of the tested extract, forms a pink-colored complex with the Griess’s reagent with an absorption maximum at 546 nm. The ability of extracts to scavenge OH^•^ formed in the Fenton reaction (c(OH^•^) = 0.7 mM) was monitored in two experimental systems utilizing degradation of 2-deoxy-D-ribose and liposomes, as models of carbohydrates and lipid biomolecules that are exposed to oxidative stress. Oxidative degradation of both of the “model” compounds leads to the formation of malondialdehyde (MDA), which reacts with thiobarbituric acid (TBA), resulting in a complex with an absorption maximum at 532 nm.

The scavenging level of the tested free radicals (%) was calculated using Equation (1):

RSC (%) = 100 × (1 − A_sample_/A_control_) (1)

All measurements in the previously described assays were performed in triplicate. To obtain a realistic perspective regarding the antioxidant potential of the analyzed herbal extracts, positive controls—propyl gallate, quercetin dihydrate, ascorbic acid, and butylated hydroxytoluene, were evaluated under the same experimental conditions.

The ability of tested extracts to reduce Fe^3+^ to Fe^2+^ was analyzed using a previously described method [17]. The reduction in ferric ions in low-pH solutions due to antioxidant activity leads to the formation of a colored complex of Fe^2+^ and 2,4,6-tripyridyl-S-triazine (TPTZ), characterized by an absorption maximum at 593 nm. The reaction mixtures contained the FRAP reagent (TPTZ dissolved in hydrochloric acid, FeCl_3_, and acetate buffer (pH = 3.6)), and different concentrations of the tested extracts. The antioxidant potential of the extracts is expressed as milligram equivalents of ascorbic acid per gram of dry extract (mg AAE/g d. e.). All measurements were performed in triplicate.

#### 2.4.2. Enzyme Inhibitory Activity

The ability of the examined extracts to inhibit MAO-A and MAO-B was determined spectrofluorimetrically [18], based on the deamination of kynuramine (used as a substrate) to 4-hydroxyquinoline. In the MAO-A inhibition test, the final enzyme and substrate concentrations in the reaction mixture were 5 μg/mL and 80 μM, respectively, while in the case of the MAO-B inhibition test, the enzyme and substrate concentrations were 10 μg/mL and 50 μM. The increasing concentrations of tested extracts were added to the reaction mixture to determine the concentration-dependent percentage of enzyme activity inhibition (I (%)). Moclobemide and selegiline were used as positive controls.

A modified Ellman method [3,14] was used to test the inhibitory effect of the studied extracts on acetylcholinesterase activity. The reaction mixture contained sodium phosphate buffer (pH = 7.2), indicator (5,5′-dithiobis-(2-nitrobenzoic acid)-DTNB with NaHCO_3_), and commercially available AChE solution (enzyme activity in the final reaction mixture was 8.15 U/L). Increasing extract concentrations were added to the reaction mixture, and a decrease in enzyme activity (I (%)) was monitored. Galantamine was used as a positive control.

An experimental procedure provided by Sigma Aldrich, with modifications described in the study by Kladar, Srđenović, Grujić, Bokić, Rat, Anačkov and Božin [16] was used to determine the potential of the tested extracts to inhibit α-amylase. The reaction mixtures contained modified starch as a substrate-indicator (starch azure), porcine α-amylase (the final enzyme activity was 0.6 U/L), and sodium phosphate buffer (pH = 7.2). Increasing concentrations of examined extracts were added to the reaction mixture, and after 10 min of incubation, the reaction was stopped by adding acetic acid (50%, *w*/*w*). After centrifugation of the test tubes (3500 rpm, 20 min), the absorbance of the supernatant was measured spectrophotometrically at 595 nm. Acarbose was used as a positive control.

Similarly, the anti-alpha-glucosidase activity of the studied extracts was analyzed according to the Sigma Aldrich method, using α-glucosidase isolated from Saccharomyces cerevisiae [19]. The reaction mixtures contained potassium phosphate buffer (pH = 6.8), glutathione solution (reduced form), enzyme α-glucosidase (7.6 U/L), and *p*-nitrophenyl-α-D-glucoside (PNP-Gluc), which was used as a substrate. After the addition of increasing concentrations of the tested extracts and incubation at 37 °C for 20 min, the reaction was stopped with Na_2_CO_3_ solution. The absorbances of the resulting solutions were measured at 400 nm, and enzyme activity inhibition (I (%)) was noted. Acarbose was used as a positive control.

The inhibitory effect of herbal extracts on the activity of studied enzymes (I (%)) was calculated according to Equation (2), and consideration that 100% of enzyme activity is available in control reaction mixtures containing distilled water instead of the tested extract.

I (%) = 100 × (1 − A/A0)(2)

where A is the absorbance of the reaction mixture containing the tested extract, and A0 is the absorbance of the control mixture. All measurements in the previously described assays were performed in triplicate.

### 2.5. Data Processing

The study’s results were organized using Microsoft Office Excel (v. 2021) in a matrix-shaped dataset with dimensions 25 × 132, which was used as input for statistical processing by Tibco Statistica (v. 13.5) software. Descriptive statistics were applied to evaluate data variability, including reporting average value, median, standard deviation, and interquartile range, while box plots were used to visualize the data distribution pattern. Univariate assessment of differences between studied groups of samples (sect. *Drosocarpium* taxa) was performed by one-way ANOVA, followed by post hoc Tukey’s test, whereas the level of statistical significance was set at *p* = 0.05. Multivariate analyses applied in the form of canonical discriminant analysis and hierarchical cluster analysis were utilized as a comprehensive approach for the identification of discrimination patterns between studied taxa. CDA, as a dimension reduction technique, decreases the number of variables used for defining the initial dataset variability by calculating novel variables (termed canonical roots) which correlate with starting variables, but are also sufficient for presenting dataset variability in a low-dimensional space and by maintaining a certain proportion of the original dataset’s discriminations.

## 3. Results and Discussion

### 3.1. Chemical Characterization of the Sect. Drosocarpium Species

Preliminary chemical characterization of collected samples included the quantification of total phenolics and flavonoids in the prepared water–alcoholic extracts. The obtained results presented in Table 1 and Table S1 indicate high abundance of both classes of mentioned compounds in analyzed samples, which is reflected through median values of total phenolics >100 mg gallic acid equivalents (GAE)/g of dry extract (d. e.) and median content of total flavonoids >20 mg quercetin equivalents (QE)/g d. e., in all analyzed species. The highest amounts of total phenolics were quantified in *H. rochelii* (164.90 ± 28.92 mg GAE/g d. e.), *H. montbretii*, *H. richerii* subsp. *grisebachii*, and *H. rumeliacum* extracts, and generally corresponded to previously published results [20,21], or were somewhat lower if considering *H. montbretii* extracts studied by Babotă, Frumuzachi, Mocan, Tămaș, Dias, Pinela, Stojković, Soković, Bădărău, and Crișan [7]. Moreover, the phenolic content of the species mentioned above was comparable to *H. perforatum* extracts [22]. Still, significantly lower phenolic abundance could be noticed for *H. barbatum* (119.57 ± 35.08 mg GAE/g d. e.) and *H. spruneri* (110.43 ± 35.70 mg GAE/g d. e.) samples. The highest amounts of total flavonoids were quantified in *H. rochelii* (46.24 ± 11.43 mg QE/g d. e.) and *H. richerii* subsp. *grisebachii* (42.00 ± 10.34 mg QE/g d. e.) samples, which was in line with previous reports [21,22]. *H. barbatum* and *H. rumeliacum* extracts contained, on average, ~33 mg QE/g d. e., while the lower amount of total flavonoids, with an average value of ~24 mg QE/g d. e., was characteristic for *H. montbretii* and *H. spruneri* samples, but also displayed significant variability within the analyzed samples.

Quantifying specific secondary metabolites using liquid chromatography (HPLC-DAD (Agilent, Waldbronn, Germany); Table 2 and Table S1) in analyzed *Hypericum* species suggests a high abundance of hypericin and hyperforin, as expected according to previous reports of phytochemical analysis of the sect. *Drosocarpium* representatives [6,8]. Hypericin is a compound that attracts much research attention regarding its biological activity. Available data suggest its antidepressant, antineoplastic, antitumor, and antiviral activities [23], while current trends project its application in medical diagnostics and therapy [24]. Phytochemical studies performed in the genus *Hypericum* highlight species from the *Drosocarpium* section and *H. perforatum* as the most significant sources of hypericin. The current study has indicated the highest content of hypericin in *H. rochelii* (738.41 ± 158.09 µg/g of dry herb (d. h.)), *H. barbatum* (689.02 ± 415.42 µg/g d. h.), and *H. richerii* subsp. *grisebachii* (575.32 ± 331.60 µg/g d. h.), which corresponds to previous reports [6,10,24,25,26], while samples of *H. rumeliacum*, *H. spruneri*, and *H. montbretii* contained considerably lower amounts of this naphthodianthrone. It is of utmost importance to highlight the high hypericin content in the previously mentioned species (*H. rochelii*, *H. barbatum*, *H. richerii* subsp. *grisebachii*), since available reports suggest significantly lower abundance of this compound in *H. perforatum* [26,27]. The highest content of hyperforin was found in *H. richerii* subsp. *grisebachii* (2020.72 ± 969.13 µg/g d. h.), whereas the other studied species contained approximately two to three times lower amounts of this phloroglucinol. Other studies [25,26] have indicated *H. richerii* subsp. *grisebachii* as a member of the *Drosocarpium* section containing high quantities of hyperforin, which are in some cases comparable to those found in *H. perforatum* [28]. The highest abundance of amentoflavone was characteristic of *H. richerii* subsp. *grisebachii* (95.58 ± 55.81 µg/g d. h.), while other studied taxa also contained this biflavonoid, in lower amounts. A study by Kakouri et al. [29] indicated that not all sect. *Drosocarpium* species contain amentoflavone, but its presence suggests a higher quantity if compared to *H. perforatum*. Quercetin and its glycoside, rutin, were present in all studied taxa, while *H. richerii* subsp. *grisebachii* samples displayed the highest quantity of both compounds, corresponding to previous reports [27,30]. Moreover, the available data [27,30] support a higher abundance of quercetin and rutin in *H. richerii* subsp. *grisebachii* when compared to *H. perforatum*. However, we must state that some studies suggest the absence of quercetin from *H. rochelii* [7] and *H. montbretii* [31] species, as well as the absence of rutin from *H. barbatum* [27] and *H. montbretii* [31]. Nevertheless, rutin has been identified as an important antioxidant, anti-inflammatory, antimicrobial, anticarcinogenic, and neuroprotective agent [32]. At the same time, quercetin jointly contributes with amentoflavone and chlorogenic acid to the anti-inflammatory potential of *Hypericum* species [4,33]. Previously conducted phytochemical studies indicate that chlorogenic acid is the most frequently reported phenolic acid in *Hypericum* species [2,34]. The highest abundance of chlorogenic acid among the studied sect. *Drosocarpium* representatives was characteristic of *H. richerii* subsp. *grisebachii* samples, while previous studies suggest increased chlorogenic acid accumulation in sect. *Drosocarpium* taxa [7,29], in amounts higher than in *H. perforatum* [27,29]. Chlorogenic acid is one of the most essential dietary phenolics characterized by notable antioxidant potential, the ability to modulate glucose and lipid metabolism, and anti-inflammatory activity [35].

#### Chemometric Approach—Chemical Characterization

**Figure 1:**
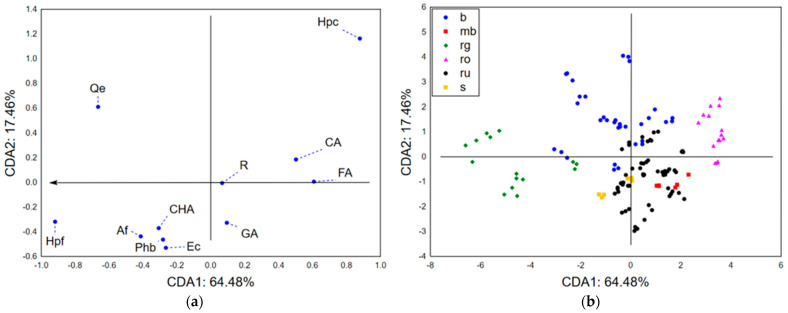
CDA-chemical characterization: (**a**) the loadings of the first two canonical roots and (**b**) the position of the analyzed samples in the space defined by the first two canonical axes. Hpc—hypericin, Hpf—hyperforin, Af—amentoflavone, R—rutin, Qe—quercetin, Ec—epicatechin, FA—ferulic acid, GA—gallic acid, CHA—chlorogenic acid, CA—caffeic acid, Phb—p-hydroxy benzoic acid, b—*H. barbatum*, mb—*H. montbretii*, rg—*H. richerii* subsp. *grisebachii*, ro—*H. rochelii*, ru—*H. rumeliacum*, and s—*H. spruneri*.

The application of canonical discriminant analysis (CDA) on a dataset summarizing the results of secondary metabolites quantification in the analyzed sect. *Drosocarpium* taxa showed that the first two canonical axes (CDA1 and CDA2) describe more than 80% of the initial dataset variability. The size of variability of analyzed samples in terms of CDA1 (Figure 1a) mostly correlated with the abundance of hypericin (Hpc), hyperforin (Hpf), and quercetin (Qe), while the shape of variability (in terms of CDA2) was predominantly defined by the content of Hpc and Qe. This emphasizes the importance of hypericin, hyperforin, and quercetin (Qe) as the most variable (in terms of the abundance) secondary metabolites in the studied sect. *Drosocarpium* species, which should be considered from several aspects. Namely, these compounds could be used as chemotaxonomic markers supporting quality control of herbal material consisting of sect. *Drosocarpium* representatives. However, considering the biological activities previously reported for hypericin, hyperforin, and quercetin, the observed variations in the abundance also suggest variability in the biological potential of these species and consequently the utilization potential. The position of analyzed samples in the space defined by the first two canonical axes (Figure 1b) shows a separative grouping of analyzed samples according to the content of quantified secondary metabolites. A compact positioning of *H. rochelii* (ro) samples in the positive part of CDA1 and CDA2 is a result of high hypericin abundance, as well as lower content of hyperforin (Hpf) and quercetin (Qe). Furthermore, joint grouping of *H. rumeliacum* (ru), *H. montbretii* (mb), and *H. spruneri* (s) samples in the negative part of CDA2 indicates mutual resemblance of their chemical profiles, which are characterized by lower abundance of Hpc, Hpf, and Qe. *H. barbatum* (b) samples are positioned in the positive and negative parts of CDA1 due to high variability in Hpc and Hpf content. Still, their positioning in the positive part of CDA2 suggests higher quercetin levels when compared to *H. rumeliacum*, *H. montbretii*, and *H. spruneri* samples. *H. richerii* subsp. *grisebachii* (rg) samples are located in the negative part of CDA1 due to the extremely high abundance of hyperforin.

**Figure 2:**
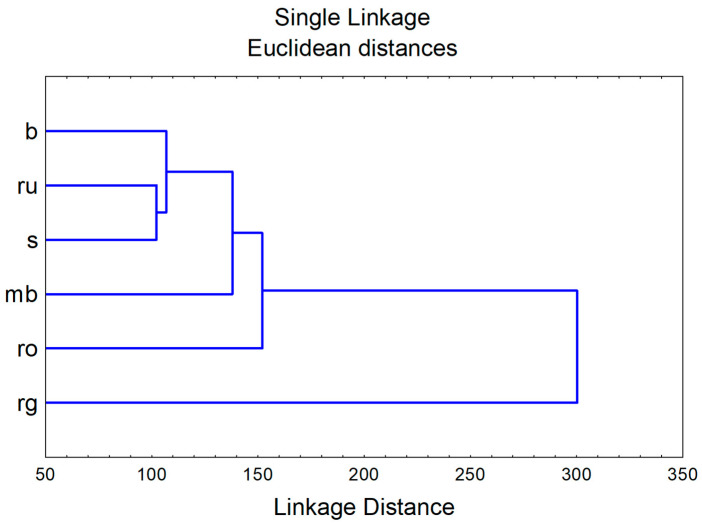
Hierarchical cluster analysis results—chemical characterization. b—*H. barbatum*, mb—*H. montbretii*, rg—*H. richerii* subsp. *grisebachii*, ro—*H. rochelii*, ru—*H. rumeliacum*, and s—*H. spruneri*.

The application of hierarchical cluster analysis on squared Mahalanobis distances obtained in CDA displays similarities between evaluated species regarding their chemical profile (Figure 2).

### 3.2. Biological Potential of the Sect. Drosocarpium Species

#### 3.2.1. Antioxidant Potential

**Figure 3:**
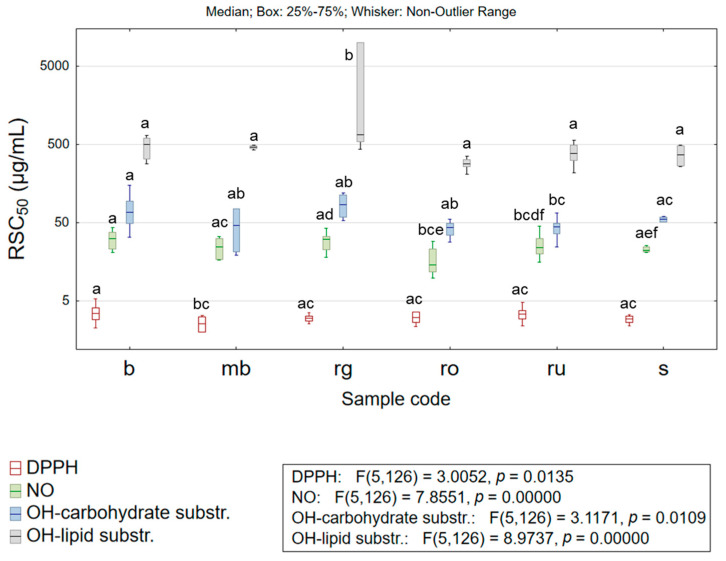
Box plots of the results describing the antioxidant potential of the studied extracts. Box plots (box—interquartile range, line—median value, whiskers—non-outlier range) were generated by considering all the results obtained for extracts of a specified taxon regarding a selected variable. The different lower-case letters denote statistically significant differences (*p* < 0.05) between evaluated species; DPPH—2,2-diphenyl-1-picrylhydrazyl radical, NO—nitroso radical, OH—hydroxyl radical, b—*H. barbatum* (n = 11 samples), mb—*H. montbretii* (n = 2 samples), rg—*H. richerii* subsp. *grisebachii* (n = 5 samples), ro—*H. rochelii* (n = 5 samples), ru—*H. rumeliacum* (n = 19 samples), and s—*H. spruneri* (n = 2 samples).

Oxidative stress and changes on the molecular level occurring as a result of uncontrolled oxidative processes play an essential role in the development of many diseases or significantly affect the course of the existing pathological conditions. Moreover, the efficacy of various antioxidant defense mechanisms is highly dependent on the dietary intake of antioxidants. At the same time, an increasing trend of demand for antioxidants of natural origin can be noticed among the human population. Numerous herbal preparations have been recognized as valuable sources of compounds displaying antioxidant activity, whereas a long tradition of their usage among the population can be used as the evidence of safe application [3]. Such findings have encouraged a trend that favors the design of functional foods with antioxidants of natural origin. The high abundance of chlorogenic acid in *Hypericum* species and its notable antioxidant potential act as a valid precondition for considering these herbs as starting material in producing functional foods [36]. However, to perform efficient screening of the antioxidant potential of the targeted agent, which is required for making valid conclusions, the application of several experimental systems is required; namely, the complexity of the organism’s oxidative processes requires evaluating several mechanisms by which potential antioxidants could display their efficacy [3]. Therefore, five experimental systems were applied to assess the antioxidant potential of the extracts studied in the current research. The obtained results are presented in Table S2 and Figure 3. Water–alcoholic extracts obtained from selected sect. *Drosocarpium* species have demonstrated strong potential to scavenge 2,2-diphenyl-1-picrylhydrazyl (DPPH) radical. Namely, the extracts’ concentrations required for scavenging of 50% of free radicals (RSC_50_ values) were <5 µg/mL, while antioxidants applied as positive controls (quercetin dihydrate and propyl gallate) in this assay demonstrated RSC_50_ of 0.97 ± 0.09 µg/mL and 0.58 ± 0.06 µg/mL, respectively. The most substantial DPPH scavenging potential was exhibited by *H. montbretii* (mb; RSC_50_ = 2.57 ± 0.59 µg/mL) and *H. spruneri* (s; RSC_50_ = 2.89 ± 0.38 µg/mL) extracts, whereas the other studied extracts displayed somewhat lower antioxidant activity. The obtained results correspond to previously conducted studies [22], but also suggest comparable antioxidant potential with *H. perforatum* [28,29]. Moreover, some studies report better DPPH radical scavenging activity of *H. richerii* subsp. *grisebachii* and *H. rochelii* extracts [7,30] when compared to *H. perforatum* [28].

Furthermore, a significant antioxidant activity related to scavenging nitroso (NO) radicals could be noticed since the mean RSC_50_ values recorded for extracts obtained from each analyzed taxon were <30 µg/mL, while propyl gallate (positive control) evaluated under the same experimental conditions demonstrated RSC_50_ = 10.12 ± 0.55 µg/mL. The significance of this finding increases if the comparison of antioxidant potential is being made with a pure substance; while herbal extracts contain numerous compounds, some of which do not act as antioxidants. The most potent NO radical scavengers were *H. rochelii* (ro) extracts with RSC_50_ = 17.42 ± 6.89 µg/mL, while the lowest antioxidant activity was characteristic for *H. barbatum* (b) and *H. richerii* subsp. *grisebachii* (rg). These findings are in agreement with previous analyses of stated species [22], and also suggest a stronger antioxidant potential if compared to *H. perforatum* [28].

The antioxidant protective effect of *Hypericum* extracts against hydroxyl (OH) radicals was studied in two complementary test systems that simulate the interaction of OH radicals with carbohydrate molecules and lipids. The obtained results suggested significantly more important antioxidant activity of the studied extracts in the case of carbohydrate protection. Namely, the average recorded RSC_50_ values obtained for the most extracts of the studied taxa were <50 µg/mL, which if compared with antioxidant effect of butylated hydroxytoluene (BHT, RSC_50_ = 0.03 ± 0.00 µg/mL), ascorbic acid (RSC_50_ = 2.70 ± 0.15 µg/mL), and propyl gallate (RSC_50_ = 11.18 ± 0.78 µg/mL) obtained under the same experimental conditions, suggests some limitations regarding OH radical scavenging potential. However, it must be stated that the *H. rochelii* (ro) and *H. rumeliacum* (ru) extracts display notable OH scavenging potential (RSC_50_ = 41.56 ± 8.07 µg/mL and RSC_50_ = 43.53 ± 11.13 µg/mL, respectively), comparable to a previous report [22], and to *H. perforatum* [28], thus exhibiting a non-negligible protective effect toward carbohydrates. On the other hand, *H. barbatum* (b) extracts seem to have low significance (RSC_50_ = 155.91 ± 47.19 µg/mL) regarding OH radicals scavenging potential. Contrary, the studied extracts are modest scavengers of OH radicals in the test system simulating the protective effect toward lipids. Namely, the average recorded RSC_50_ values for studied taxa were >250 µg/mL, which, if compared with the antioxidant potential of the positive control (BHT, RSC_50_ = 6.55 ± 0.42 µg/mL), confirms the limiting antioxidant protective effects toward lipids.

The potential of studied extracts to reduce ferric ion (FRAP assay) indicated strong antioxidant activity since all analyzed taxa displayed an average value >140 mg ascorbic acid equivalents (AAEs)/g d. e., while the best activity in the applied assay was recorded for *H. rumeliacum* (ru; 191.48 ± 41.87 mg AAE/g d. e.) and *H. rochelii* (ro; 184.15 ± 40.19 mg AAE/g d. e.) extracts. These findings correspond to previous reports [22,27], but also suggest comparable antioxidant potential with *H. perforatum* [27,28].

#### 3.2.2. Inhibition of Biologically Important Enzymes

**Figure 4:**
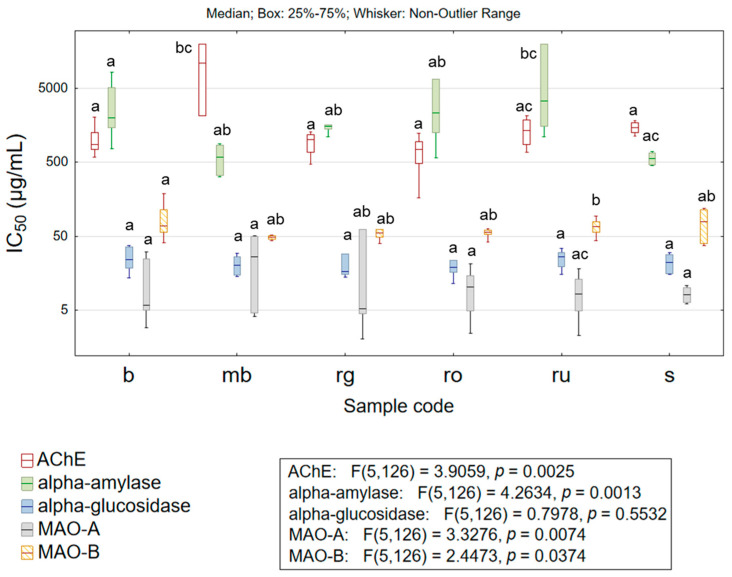
Box plots of the results describing the enzyme inhibitory potential of the studied extracts. Box plots (box—interquartile range, line—median value, whiskers—non-outlier range) were generated by considering all the results obtained for extracts of the specified taxon regarding the selected variable. The different lower-case letters denote statistically significant differences (*p* < 0.05) between evaluated species; AChE—acetylcholinesterase, MAO-A—monoamine oxidase A, MAO-B—monoamine oxidase B, b—*H. barbatum* (n = 11 samples), mb—*H. montbretii* (n = 2 samples), rg—*H. richerii* subsp. *grisebachii* (n = 5 samples), ro—*H. rochelii* (n = 5 samples), ru—*H. rumeliacum* (n = 19 samples), and s—*H. spruneri* (n = 2 samples).

Modulation of enzyme activity has been recognized as a valid pharmacological approach in the treatment of many diseases. The example of enzymes of which inhibition is clinically significant is acetylcholinesterase (AChE) or monoamine oxidases (MAO-A and MAO-B). Namely, the reduction in AChE activity is used in the treatment of Alzheimer’s disease, Parkinson’s disease, and Lewy body dementia [37], while MAO inhibition is significant in the treatment of depression and Parkinson’s disease [22]. Although agents displaying AChE or MAO inhibitory effects are being utilized for the treatment of the aforementioned clinical conditions, their adverse effects keep the research on novel molecules still in focus. The potential of the studied herbal extracts to inhibit some of the biologically important enzymes is presented in Table S2 and Figure 4.

The obtained results suggest a modest anticholinesterase effect. Specifically, the comparison between the most active extracts of *H. richerii* subsp. *grisebachii* (rg) and *H. rochelii* (ro), which inhibited 50% of enzyme activity in concentrations IC_50_ = 922.01 ± 284.87 µg/mL and IC_50_ = 701.89 ± 355.15 µg/mL, respectively, and galantamine (positive control, IC_50_ = 8.32 ± 0.537 µg/mL) clearly depicts the low significance of herbal extracts’ activity. However, it is worth mentioning that these extracts show similar anticholinesterase potential as *H. perforatum* [7,28]. Moreover, the evaluated extracts have displayed low anti-MAO-B potential. The average IC_50_ value recorded for extracts of each studied taxon was >50 µg/mL, while selegiline—the positive control, inhibited enzyme activity at IC_50_ = 0.27 ± 0.03 µg/mL. On the other hand, more encouraging results regarding MAO-A inhibition have been obtained. Namely, the extracts of *H. rochelii* (ro), *H. rumeliacum* (ru), and *H. spruneri* (s) displayed anti-MAO-A activity with an average IC_50_ value of ~10 µg/mL, which can be considered as comparable to the inhibitory effect of moclobemide (IC_50_ = 0.84 ± 0.09 µg/mL), since the comparison is being made between pure compound and complex mixture (herbal extract) containing a significant share of substances with no activity. The obtained results were in agreement with previous findings [22] and at the same time have suggested a resemblance in anti-MAO-A potential with *H. perforatum* extracts [28].

The decrease in α-amylase and α-glucosidase activity reduces postprandial glycaemia and hyperinsulinemia, which is of utmost importance for successful clinical management of diabetes mellitus type 2 [3,38]. The studied herbal extracts have displayed modest anti-α-amylase potential, whereas the highest inhibitory activity was characteristic for *H. montbretii* (mb; IC_50_ = 593.89 ± 284.23 µg/mL) and *H. spruneri* (s; IC_50_ = 566.20 ± 116.99 µg/mL) extracts. The practical significance of reported activity can be evaluated by comparison with the anti-α-amylase potential of acarbose (IC_50_ = 4.99 ± 0.37 µg/mL), evaluated under the same experimental conditions. On the other hand, water–alcoholic extracts obtained from the studied *Hypericum* species exhibited notable α-glucosidase inhibitory activity. Specifically, the extracts obtained from each of the studied taxa inhibited α-glucosidase with an average IC_50_ < 30 µg/mL, while the acarbose (positive control) activity was IC_50_ = 52.14 ± 3.87 µg/mL. Moreover, although no taxon-related statistically significant differences were found in the anti-α-glucosidase activity of extracts, it is worth mentioning that some of the analyzed samples inhibited α-glucosidase with IC_50_ < 15 µg/mL (Table S2). The obtained results were generally in accordance with previous studies [7,22], but also suggest similar [28] or stronger anti-α-glucosidase activity of sect. *Drosocarpium* species when compared to *H. perforatum* [7,28]. The notable antihyperglycemic potential of sect. *Drosocarpium* species, assisted by strong antioxidant activity, suggests these extracts as a valuable resource in the production of functional foods displaying health-promoting effects in patients with metabolic syndrome or type 2 diabetes.

#### 3.2.3. Chemometric Approach—Biological Potential

**Figure 5:**
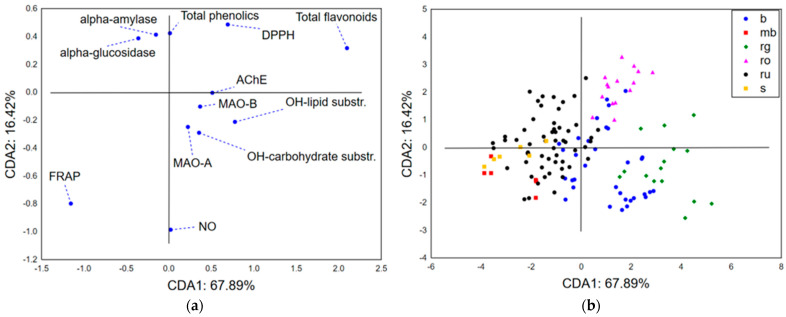
CDA-biological potential: (**a**) the loadings of the first two canonical roots and (**b**) the position of the analyzed samples in the space defined by the first two canonical axes. DPPH—2,2-diphenyl-1-picrylhydrazyl radical, NO—nitroso radical, OH—hydroxyl radical, FRAP—ferric reducing antioxidant potential, AChE—acetylcholinesterase, MAO-A—monoamine oxidase A, MAO-B—monoamine oxidase B, b—*H. barbatum*, mb—*H. montbretii*, rg—*H. richerii* subsp. *grisebachii*, ro—*H. rochelii*, ru—*H. rumeliacum*, and s—*H. spruneri*.

The application of CDA on the results of biological potential evaluation combined with total phenolics and flavonoids quantification indicates that the first two canonical axes describe more than 84% of the initial discriminations. The size of recorded discriminations primarily correlates with the content of total flavonoids and results of antioxidant potential evaluated by the activity of ferric ion reduction (Figure 5a). On the other hand, the shape of dataset discriminations (in terms of CDA2) is correlated with the scavenging capacity of NO and DPPH radicals, and also with total phenolics content and extracts’ antihyperglycemic potential (inhibition of α-amylase and α-glucosidase). The position of the analyzed samples in the space defined by CDA1 and CDA2 (Figure 5b) suggests close grouping of *H. rochelii* (ro) samples in the positive part of CDA1 and CDA2, due to the samples being abundant in flavonoids and phenolics, having lower DPPH radical (higher RSC_50_ values), and notable NO radical scavenging potential, as well as displaying lower anti-α-glucosidase activity (higher IC_50_ values). On the contrary, *H. richerii* subsp. *grisebachii* (rg) samples are primarily positioned in the positive part of CDA1 and negative part of CDA2 based on the lower content of total phenolics, stronger scavenging potential of DPPH radicals, less prominent scavenging of NO radicals, and notable antihyperglycemic activity. The negative part of CDA1 shows grouping of *H. rumeliacum* (ru), *H. montbretii* (mb), and *H. spruneri* (s) samples. *H. rumeliacum* samples suggest a lower abundance of flavonoid compounds and promising antioxidant potential recorded in the FRAP assay. Still, they are also characterized by a pattern of high variability related to their antihyperglycemic potential, total phenolics content, and NO radical scavenging capacity. On the other hand, *H. spruneri* and *H. montbretii* samples are closely grouped in the negative part of CDA1 and CDA2 as a consequence of lower content of total phenolics and flavonoids, significant antihyperglycemic activity, and DPPH radical scavenging potential.

## 4. Conclusions

The conducted research indicates a high abundance of secondary metabolites characteristic for the genus *Hypericum* among the sect. *Drosocarpium* representatives, thus supporting previous reports on the application of some of these species in traditional medicine. Specifically, higher content of hypericin (in species *H. rochelii*, *H. barbatum*, and *H. richerii* subsp. *grisebachii*), amentoflavone (in *H. richerii* subsp. *grisebachii*), quercetin, and rutin (in *H. richerii* subsp. *grisebachii*), as well as chlorogenic acid (in *H. richerii* subsp. *grisebachii*, *H. barbatum*, *H. rumeliacum*), was found when compared to *H. perforatum*. At the same time, the other evaluated taxa usually contained similar amounts of secondary metabolites as *H. perforatum*.

The water–alcoholic extracts of the studied species were highly active scavengers of free radicals, especially DPPH and NO radicals, as well as OH radicals in an assay evaluating protective effects toward carbohydrate molecules, while the recorded antioxidant activity was frequently higher in comparison to *H. perforatum* extracts. Also, the studied extracts were potent inhibitors of α-glucosidase and MAO-A, often more active than *H. perforatum* extracts. These findings suggest the potential of having multiple beneficial effects in chronic diseases, such as diabetes mellitus and some neurodegenerative diseases, of which specific treatment includes modulation of α-glucosidase and MAO-A activity, but also nonspecific approaches for boosting organisms’ antioxidant defense. Therefore, the results of phytochemical profiling and biological potential evaluation that have been obtained provide us with strong foundations for further research on the sect. *Drosocarpium* species and allow a justified hypothesis on these species as a valuable resource for designing functional foods and medicines. However, the proposed research direction should be at the same time followed by studies on sustainable production of stated species in order to maintain their current biodiversity.

## Figures and Tables

**Table 1:** The results of preliminary chemical characterization of the sect. *Drosocarpium* species.

Taxon	*H. barbatum* (n = 11 Samples)			*H. montbretii* (n = 2 Samples)			*H. richerii* subsp. *grisebachii* (n = 5 Samples)			*H. rochelii* (n = 5 Samples)			*H. rumeliacum* (n = 19 Samples)			*H. spruneri* (n = 2 Samples)		
	MV	Mdn	SD	MV	Mdn	SD	MV	Mdn	SD	MV	Mdn	SD	MV	Mdn	SD	MV	Mdn	SD
Total phenolics (mg GAE/g d. e.)	119.57 ^a^	119.42	35.08	152.25 ^ac^	149.99	66.56	142.71 ^ad^	141.28	31.76	164.90 ^bcd^	166.95	28.92	145.31 ^bcde^	142.97	29.49	110.43 ^ae^	109.62	35.70
Total flavonoids (mg QE/g d. e.)	33.76 ^a^	34.19	11.11	25.43 ^a^	25.49	2.86	44.92 ^b^	42.00	10.34	46.24 ^b^	47.86	11.43	33.27 ^a^	32.25	7.70	22.91 ^a^	23.19	2.50
Dry extract yield (%)	14.43	15.25	3.03	19.21	19.08	1.84	17.48	18.45	2.96	18.46	19.08	3.62	16.64	16.30	3.28	19.05	18.71	2.11

The results are expressed as mean (MV) and median (Mdn) values, and standard deviation (SD) of results obtained for a specific variable, considering all analyzed samples of a particular taxon. The different lower-case letters denote statistically significant differences (*p* < 0.05) between evaluated species; d. e.—dry extract, GAE—gallic acid equivalents, QE—quercetin equivalents.

**Table 2:** Secondary metabolites of the sect. *Drosocarpium* species.

	*H. barbatum* (n = 11 Samples)			*H. montbretii* (n = 2 Samples)			*H. richerii* subsp. *grisebachii* (n = 5 Samples)			*H. rochelii* (n = 5 Samples)			*H. rumeliacum* (n = 19 Samples)			*H. spruneri* (n = 2 Samples)		
	MV	Mdn	SD	MV	Mdn	SD	MV	Mdn	SD	MV	Mdn	SD	MV	Mdn	SD	MV	Mdn	SD
	µg/g of dry herb																	
Hpc	689.02 ^a^	573.44	415.42	215.42 ^bc^	208.45	159.65	575.32 ^acde^	560.54	331.60	738.41 ^a^	774.12	158.09	366.60 ^bd^	291.16	229.74	311.49 ^be^	308.69	80.87
Hpf	998.65 ^a^	953.08	481.14	311.76 ^bc^	309.31	196.65	2020.72 ^b^	1773.03	969.13	702.84 ^ac^	630.59	181.10	487.28 ^bc^	446.31	223.66	833.70 ^ac^	823.17	237.88
Af	51.65 ^a^	45.14	43.87	27.36 ^a^	26.75	6.12	95.58 ^bc^	120.37	55.81	28.49 ^a^	29.72	21.41	47.14 ^a^	51.03	26.50	67.21 ^ac^	65.61	36.33
R	216.29 ^a^	147.81	152.05	115.99 ^ac^	114.87	77.75	812.08 ^b^	784.73	405.48	97.86 ^ac^	109.72	19.93	208.03 ^ac^	100.60	331.07	110.05 ^ac^	107.39	24.56
Qe	148.52 ^a^	163.62	60.87	89.59 ^bc^	86.52	66.88	210.25 ^b^	194.44	55.00	32.75 ^bcd^	n.d.	41.60	78.06 ^bc^	73.18	33.43	75.42 ^bcd^	76.52	8.57
Ec	32.64 ^a^	n.d.	71.82	1268.07 ^b^	1207.34	1391.63	131.42 ^a^	n.d.	272.52	95.82 ^a^	n.d.	198.56	223.19 ^a^	n.d.	482.77	n.d. ^a^	n.d.	/
FA	6.82 ^a^	n.d.	21.87	304.14 ^b^	290.64	260.18	n.d. ^a^	n.d.	/	247.00 ^b^	264.04	233.38	143.84 ^bc^	96.22	180.11	n.d. ^ac^	n.d.	/
GA	50.90 ^a^	46.35	47.55	n.d. ^a^	n.d.	/	111.14 ^bc^	127.13	41.54	40.55 ^a^	34.22	40.89	55.55 ^a^	30.10	75.43	73.75 ^ac^	73.22	24.37
CHA	227.78 ^a^	167.32	228.34	21.75 ^a^	20.85	23.84	655.57 ^b^	205.51	618.24	94.00 ^a^	114.19	87.96	192.93 ^a^	140.52	309.37	178.42 ^a^	180.63	12.31
CA	62.52 ^a^	62.50	24.49	106.68 ^ab^	103.02	100.18	69.88 ^a^	58.65	28.55	88.39 ^ab^	93.15	37.85	51.66 ^ac^	48.89	45.42	71.02 ^a^	69.52	15.04
Phb	293.86 ^a^	118.34	351.90	258.30 ^ad^	256.17	84.47	124.94 ^abd^	105.03	74.44	208.26 ^ac^	288.42	152.69	1187.06 ^bcd^	946.61	1976.97	923.92 ^ad^	898.64	284.56

The results are expressed as mean (MV) and median (Mdn) values, and standard deviation (SD) of results obtained for a specific variable, considering all analyzed samples of a particular taxon. The different lower-case letters denote statistically significant differences (*p* < 0.05) between evaluated species; n.d.—not detected, Hpc—hypericin, Hpf—hyperforin, Af—amentoflavone, R—rutin, Qe—quercetin, Ec—epicatechin, FA—ferulic acid, GA—gallic acid, CHA—chlorogenic acid, CA—caffeic acid, Phb—p-hydroxy benzoic acid.

## Data Availability

All of the relevant data are available within the manuscript or as submitted Supplementary Materials.

## Supplementary Materials

The following supporting information can be downloaded at https://www.mdpi.com/article/10.3390/life15081332/s1; Table S1: The results of chemical characterization of analyzed samples; Table S2: The results of the evaluation of the biological potential of the analyzed extracts; Table S3: Herbal samples used in the research.
