# Supplementary material for: Biological Potential of Hypericum L. Sect. Drosocarpium Species
Source: Life (Basel). 2025 Aug 21;15(8):1332. doi: 10.3390/life15081332 (PMC12387250; doi:10.3390/life15081332)
Supplement: Supplementary file 1 [file life-15-01332-s001.zip › Proof_corrected_Suppl/Table S1_v1.pdf]

Table S1. The results of chemical characterization of the analyzed samples expressed as an average value and standard deviation (SD) of three repeated measurements (n=3). The quantities are given per dry extract (d.e.) or dried herb. GAE-gallic acid equivalents, QE-quercetin equivalents, Hpc-hypericin, Hpf-hyperforin, Af-amentoflavone, R-rutin, Qe-quercetin, Ec-epicatechin, FA-ferulic acid, GA-gallic acid, CHA-chlorogenic acid, CA-caffeic acid, Phb-p-hydroxy benzoic acid, n.d.-not detected.

			Preliminary chemical characterization						Quantification (µg/g of dried herb) of specific secondary metabolites (HPLC-DAD)																					
Taxon	Sample	Sample code	Total phenolics (mg GAE/g d.e.)		Total flavonoids (mg QE/g d.e.)		Dry extract yield (%)		Hpc		Hpf		Af		R		Qe		Ec		FA		GA		CHA		CA		Phb	
			Mean	SD	Mean	SD	Mean	SD	Mean	SD	Mean	SD	Mean	SD	Mean	SD	Mean	SD	Mean	SD	Mean	SD	Mean	SD	Mean	SD	Mean	SD	Mean	SD
H. barbatum	b1	b	50.63	1.86	15.48	0.10	15.25	0.33	619.11	27.48	601.23	10.97	n.d.	n.d.	431.11	18.00	90.50	0.30	n.d.	n.d.	n.d.	n.d.	74.87	2.34	106.03	0.40	64.68	1.89	58.53	2.78
H. barbatum	b2	b	138.54	2.92	38.73	0.82	11.85	0.81	612.60	15.56	1216.79	2.35	n.d.	n.d.	145.79	3.10	237.86	11.13	n.d.	n.d.	n.d.	n.d.	26.81	0.10	187.86	1.13	38.37	0.61	118.74	5.40
H. barbatum	b3	b	170.40	8.26	34.62	1.67	6.66	0.38	402.18	8.13	454.97	1.65	38.06	1.07	56.06	1.59	151.07	5.33	n.d.	n.d.	n.d.	n.d.	n.d.	n.d.	105.01	4.18	28.65	1.16	66.12	2.83
H. barbatum	b4	b	142.87	5.10	41.69	0.99	16.23	0.12	225.11	10.16	1412.60	60.38	41.71	1.22	436.90	9.22	220.35	4.50	n.d.	n.d.	n.d.	n.d.	144.98	5.57	150.39	4.95	102.61	0.80	57.60	2.35
H. barbatum	b5	b	93.50	0.62	32.38	1.04	15.05	0.02	1098.00	17.41	1854.43	19.90	135.46	0.07	316.16	9.44	193.66	6.41	n.d.	n.d.	n.d.	n.d.	112.82	0.81	154.03	5.94	102.29	1.38	117.45	2.54
H. barbatum	b6	b	140.21	4.65	41.60	1.42	15.03	0.19	1435.50	69.96	1560.53	68.21	105.57	4.46	448.39	5.39	59.52	0.77	n.d.	n.d.	n.d.	n.d.	85.17	4.18	930.68	15.73	54.62	2.65	194.85	9.59
H. barbatum	b7	b	95.85	0.14	20.99	0.34	15.07	0.63	310.68	7.51	338.32	16.16	59.60	1.12	70.45	2.91	177.60	4.25	n.d.	n.d.	79.13	2.30	13.04	0.51	137.51	2.23	82.75	2.94	369.96	16.83
H. barbatum	b8	b	173.09	3.28	52.84	0.88	17.47	0.86	363.84	8.96	653.86	23.66	51.60	1.09	75.72	3.67	89.50	0.22	n.d.	n.d.	n.d.	n.d.	n.d.	n.d.	205.15	4.03	40.18	0.47	1084.64	13.56
H. barbatum	b9	b	102.46	1.60	31.09	0.41	16.84	0.45	735.34	26.33	718.19	22.05	88.98	3.46	106.94	0.15	76.71	3.26	144.81	3.26	n.d.	n.d.	7.80	0.13	170.75	2.44	69.74	0.10	921.41	36.94
H. barbatum	b10	b	119.42	4.23	41.62	1.89	12.63	0.68	445.80	8.23	896.93	8.93	n.d.	n.d.	173.67	2.67	166.22	1.00	n.d.	n.d.	n.d.	n.d.	46.48	2.11	187.23	8.01	39.81	0.11	118.34	4.96
H. barbatum	b11	b	97.42	4.68	20.89	0.28	16.23	0.51	1287.98	60.23	1520.57	53.90	45.37	1.24	151.15	2.40	163.62	4.40	220.36	5.36	n.d.	n.d.	48.00	0.80	130.39	4.18	67.23	0.77	112.79	1.76
H. montbretii	mb1	mb	210.36	8.71	29.02	0.42	17.53	0.02	359.83	13.09	507.71	16.22	31.17	0.76	183.68	4.09	143.36	1.56	2515.72	41.63	508.38	10.00	n.d.	n.d.	n.d.	n.d.	189.62	1.72	320.19	15.15
H. montbretii	mb2	mb	89.83	3.55	24.28	0.53	20.71	0.61	68.73	1.50	133.12	3.82	22.32	1.11	46.07	1.50	29.69	0.52	n.d.	n.d.	61.98	2.36	n.d.	n.d.	44.12	1.04	14.71	0.04	171.86	2.64
H. richerii subsp. *grisebachii*	rg1	rg	133.24	2.65	45.00	0.02	18.46	0.30	334.66	7.78	1773.03	40.02	153.73	5.77	784.73	28.16	194.44	5.86	n.d.	n.d.	n.d.	n.d.	127.13	4.76	200.34	4.87	51.92	1.25	265.35	5.47
H. richerii subsp. *grisebachii*	rg2	rg	173.32	2.61	57.67	1.88	12.25	0.45	150.74	3.94	853.38	9.58	n.d.	n.d.	206.53	4.69	161.35	3.22	616.56	2.84	n.d.	n.d.	33.44	0.20	166.28	1.66	40.29	0.13	105.03	1.94
H. richerii subsp. *grisebachii*	rg3	rg	172.97	8.02	53.18	0.62	17.34	0.44	613.91	19.45	1706.70	16.74	132.00	2.89	1311.99	31.18	218.56	9.28	n.d.	n.d.	n.d.	n.d.	121.81	3.66	1175.81	9.09	83.02	2.68	104.77	4.59
H. richerii subsp. *grisebachii*	rg4	rg	119.25	0.49	39.10	1.80	18.46	0.96	1090.56	53.59	3577.70	124.50	77.45	0.07	628.58	4.08	174.84	5.45	n.d.	n.d.	n.d.	n.d.	122.98	5.81	190.99	8.23	59.71	2.55	87.41	0.43
H. richerii subsp. *grisebachii*	rg5	rg	107.31	0.05	31.33	1.46	20.77	0.44	687.71	3.43	2140.68	51.55	117.43	0.06	1122.49	10.84	290.61	11.94	n.d.	n.d.	n.d.	n.d.	145.40	0.72	1713.42	60.84	108.90	1.13	58.05	1.47
H. rochelii	ro1	ro	187.84	0.15	62.29	2.49	18.09	0.72	456.18	21.03	647.57	11.49	17.11	0.76	69.01	0.39	n.d.	n.d.	n.d.	n.d.	418.78	3.11	10.25	0.17	n.d.	n.d.	44.20	0.18	344.14	10.17
H. rochelii	ro2	ro	156.72	6.26	26.71	0.92	19.08	0.30	894.99	41.60	630.59	19.62	30.52	0.64	109.72	1.75	n.d.	n.d.	500.24	11.02	n.d.	n.d.	1.55	0.07	128.63	3.19	130.90	0.42	268.53	11.04
H. rochelii	ro3	ro	161.57	7.06	50.32	0.23	12.28	0.33	767.05	11.90	1052.18	8.83	n.d.	n.d.	75.08	0.02	87.00	0.47	n.d.	n.d.	553.14	21.43	37.25	1.39	n.d.	n.d.	47.72	0.28	58.81	1.24
H. rochelii	ro4	ro	119.65	2.70	44.49	1.95	19.67	0.03	819.78	11.52	670.64	6.34	59.38	2.96	114.67	3.79	74.96	0.46	n.d.	n.d.	274.22	8.61	43.32	0.71	126.59	2.58	112.17	2.22	356.99	8.21
H. rochelii	ro5	ro	192.93	4.53	50.12	2.03	22.64	0.08	867.86	2.85	568.73	8.22	33.22	0.73	123.15	2.51	n.d.	n.d.	n.d.	n.d.	n.d.	n.d.	121.05	3.53	231.96	2.90	98.59	3.39	n.d.	n.d.
H. rumeliacum	ru1	ru	188.68	6.43	42.74	0.80	16.15	0.85	76.57	3.33	178.78	2.11	25.33	0.33	55.34	0.42	62.11	1.52	n.d.	n.d.	n.d.	n.d.	n.d.	n.d.	114.60	4.96	41.22	1.58	72.21	2.57
H. rumeliacum	ru2	ru	183.54	5.26	33.01	0.10	15.86	0.40	585.16	0.87	444.23	19.62	32.55	0.52	84.85	3.86	62.20	1.90	113.79	1.18	n.d.	n.d.	9.89	0.02	166.63	0.08	n.d.	n.d.	n.d.	n.d.
H. rumeliacum	ru3	ru	149.82	0.51	27.53	0.09	13.28	0.42	145.85	1.21	349.32	6.02	15.34	0.39	254.76	10.64	73.78	0.41	46.09	1.05	n.d.	n.d.	32.63	0.03	535.48	26.21	17.17	0.30	42.11	1.53
H. rumeliacum	ru4	ru	153.97	7.21	32.20	0.51	17.47	0.68	175.56	3.27	564.46	15.67	n.d.	n.d.	122.51	1.12	88.49	3.54	1650.58	69.92	153.23	1.12	63.20	2.18	n.d.	n.d.	58.75	0.79	212.45	5.21
H. rumeliacum	ru5	ru	160.49	7.70	44.37	0.97	13.10	0.70	526.36	22.88	449.47	3.81	60.14	2.06	105.29	3.82	65.01	0.70	n.d.	n.d.	n.d.	n.d.	44.86	1.84	231.19	2.33	n.d.	n.d.	1277.67	31.83
H. rumeliacum	ru6	ru	155.12	7.58	38.92	0.11	11.95	0.28	224.37	1.61	752.78	13.05	46.43	2.09	n.d.	n.d.	74.48	2.63	1421.19	33.32	457.18	10.47	30.10	1.21	204.75	2.14	62.65	0.81	1185.44	10.45
H. rumeliacum	ru7	ru	170.40	2.81	32.55	0.99	18.14	0.72	487.11	10.68	560.53	21.19	37.49	1.42	111.12	5.07	63.98	2.42	n.d.	n.d.	227.37	5.08	29.55	0.22	154.38	0.39	85.93	3.32	946.61	9.07
H. rumeliacum	ru8	ru	109.67	3.19	26.26	0.16	17.28	0.51	622.65	10.22	511.04	4.23	87.25	0.37	97.68	4.24	64.31	2.62	299.25	8.18	96.22	2.86	37.28	0.03	n.d.	n.d.	n.d.	n.d.	1107.66	47.67
H. rumeliacum	ru9	ru	151.75	5.04	32.70	0.32	19.93	0.40	517.87	21.86	396.02	4.30	69.62	1.20	78.05	2.39	123.90	0.25	n.d.	n.d.	n.d.	n.d.	36.99	1.02	225.30	2.80	154.32	3.79	1054.89	46.73
H. rumeliacum	ru10	ru	127.46	2.50	35.46	0.02	15.26	0.94	278.55	11.58	353.63	5.10	65.82	2.48	102.79	1.14	145.51	6.11	125.86	3.29	697.83	5.87	320.19	2.45	187.14	7.40	49.17	0.55	995.86	1.55
H. rumeliacum	ru11	ru	145.68	3.70	34.49	0.16	15.75	0.35	299.76	5.55	749.95	27.92	56.46	2.54	1123.35	51.32	111.42	4.72	n.d.	n.d.	n.d.	n.d.	192.05	7.76	204.91	3.80	66.85	1.03	72.46	0.05
H. rumeliacum	ru12	ru	139.45	3.96	33.20	0.85	19.65	0.58	567.25	15.48	508.55	20.95	46.12	2.12	76.88	1.09	90.65	3.20	n.d.	n.d.	325.92	15.98	24.29	0.41	175.10	4.07	100.25	2.01	9146.52	9.93
H. rumeliacum	ru13	ru	196.75	6.09	51.52	0.71	27.32	1.41	706.42	4.68	1085.60	38.91	65.63	0.45	128.05	2.41	118.55	5.73	n.d.	n.d.	114.57	1.13	92.18	0.04	n.d.	n.d.	73.09	0.13	2100.63	13.55
H. rumeliacum	ru14	ru	114.55	1.98	22.59	0.16	15.97	0.18	100.23	0.60	197.43	6.58	23.76	0.81	105.53	2.52	85.05	0.47	n.d.	n.d.	n.d.	n.d.	104.91	0.29	n.d.	n.d.	121.16	0.78	610.57	25.92
H. rumeliacum	ru15	ru	130.15	3.53	32.25	1.45	17.94	0.87	183.37	1.90	311.90	9.77	56.92	0.45	93.18	2.29	68.72	0.95	83.84	2.55	n.d.	n.d.	23.51	0.11	n.d.	n.d.	n.d.	n.d.	1040.07	30.50
H. rumeliacum	ru16	ru	80.55	3.97	21.32	0.34	14.30	0.03	88.48	4.05	163.67	4.83	51.01	2.39	78.71	1.28	75.41	3.36	n.d.	n.d.	180.99	6.41	n.d.	n.d.	107.45	4.02	126.15	6.03	490.77	7.50
H. rumeliacum	ru17	ru	171.72	2.58	34.40	0.97	13.88	0.43	897.49	15.51	760.58	9.75	104.42	3.14	1079.51	4.52	47.08	0.68	115.37	3.40	n.d.	n.d.	11.05	0.35	1306.28	55.11	15.78	0.58	54.77	2.49
H. rumeliacum	ru18	ru	115.33	5.01	28.83	1.20	16.30	0.80	398.67	14.19	621.93	4.34	56.52	2.16	101.97	1.27	n.d.	n.d.	n.d.	n.d.	305.17	12.12	n.d.	n.d.	n.d.	n.d.	n.d.	n.d.	1266.66	19.05
H. rumeliacum	ru19	ru	133.95	1.52	27.06	1.10	16.41	0.92	208.86	0.42	427.37	19.25	n.d.	n.d.	64.48	2.86	55.77	0.09	299.44	6.48	217.89	3.85	15.90	0.22	n.d.	n.d.	30.76	0.75	824.70	9.17
H. spruneri	s1	s	76.42	0.90	21.02	0.06	17.53	1.10	254.41	11.94	620.82	23.91	36.94	0.91	89.04	2.69	68.65	2.30	n.d.	n.d.	n.d.	n.d.	54.74	0.49	158.05	0.41	59.72	0.39	651.30	4.27
H. spruneri	s2	s	150.62	5.35	26.44	1.03	20.79	1.07	399.96	8.17	994.27	41.53	106.22	3.51	125.74	0.71	83.92	1.43	n.d.	n.d.	n.d.	n.d.	97.41	2.96	179.26	1.83	86.28	4.03	1220.69	35.07
