# Supplementary material for: Biological Potential of Hypericum L. Sect. Drosocarpium Species
Source: Life (Basel). 2025 Aug 21;15(8):1332. doi: 10.3390/life15081332 (PMC12387250; doi:10.3390/life15081332)
Supplement: Supplementary file 1 [file life-15-01332-s001.zip › Proof_corrected_Suppl/Table S2_v2_Corr.pdf]

Table S2. The results of the evaluation of the biological potential of the analyzed extracts are expressed as an average value and standard deviation (SD) of three repetitive measurements (n=3). DPPH-2,2-diphenyl-1-picrylhydrazyl radical, NO-nitroso radical, OH-hydroxyl radical, FRAP-ferric reduction antioxidant potential, AAE-ascorbic acid equivalents, AChE-acetylcholinesterase, MAO-A-monoamine oxidase A, MAO-B-monoamine oxidase B, d.e.-dry extract, n.d.-not detected.

Taxon	Sample	Sample code	Antioxidant potential										Inhibition of biologically important enzymes									
			DPPH (RSC_{50}, µg/mL)		NO (RSC_{50}, µg/mL)		OH (RSC_{50}, µg/mL) carbohydrate substrate		OH (RSC_{50}, µg/mL) lipid substrate		FRAP (mg AAE/g d.e.)		AChE (IC_{50}, µg/mL)		α-amylase (IC_{50}, µg/mL)		α-glucosidase (IC_{50}, µg/mL)		MAO-A (IC_{50}, µg/mL)		MAO-B (IC_{50}, µg/mL)	
			Mean	SD	Mean	SD	Mean	SD	Mean	SD	Mean	SD	Mean	SD	Mean	SD	Mean	SD	Mean	SD	Mean	SD
H. barbatum	b1	b	3.43	0.14	37.26	2.73	94.45	8.51	648.51	18.44	71.93	7.00	n. d.	n. d.	2740.84	64.36	37.11	1.24	5.02	0.12	186.81	5.82
H. barbatum	b2	b	2.92	0.05	22.87	2.24	51.31	1.43	317.23	26.30	185.07	11.37	1265.50	43.48	1106.86	31.59	13.52	0.61	5.27	0.08	69.12	0.41
H. barbatum	b3	b	5.32	0.02	32.47	3.07	48.77	2.42	601.87	51.96	180.40	2.48	998.58	53.70	1700.67	50.88	33.79	0.09	6.94	0.51	120.30	4.96
H. barbatum	b4	b	3.45	0.11	36.40	0.08	36.10	1.06	503.92	30.17	198.01	3.77	837.33	21.59	n. d.	n. d.	15.99	0.85	3.12	0.30	52.59	2.47
H. barbatum	b5	b	2.77	0.13	22.43	0.80	n. d.	n. d.	348.90	19.80	131.00	8.41	721.80	17.33	1538.74	27.35	24.98	0.87	4.06	0.09	75.75	2.84
H. barbatum	b6	b	3.61	0.08	31.46	2.88	51.60	1.60	548.15	19.97	133.36	8.51	1343.56	35.74	5110.66	234.93	87.47	4.59	4.43	0.26	52.41	0.62
H. barbatum	b7	b	4.21	0.19	42.61	1.60	38.09	2.68	n. d.	n. d.	88.73	4.50	731.58	25.35	7992.00	340.77	37.53	1.45	30.84	0.72	102.64	5.47
H. barbatum	b8	b	2.66	0.07	22.90	0.36	67.67	5.38	282.60	3.96	228.94	20.20	2228.57	18.69	825.13	26.97	18.60	0.21	208.67	9.03	60.00	2.73
H. barbatum	b9	b	3.74	0.09	22.92	1.89	139.93	13.24	544.66	29.76	142.62	13.04	741.87	27.07	2221.82	41.63	23.68	0.68	6.44	0.07	55.83	5.16
H. barbatum	b10	b	2.96	0.05	32.58	0.26	91.18	1.38	315.46	10.84	125.33	9.96	606.43	27.56	1506.34	44.27	20.27	0.07	7.17	0.45	40.24	1.64
H. barbatum	b11	b	4.04	0.03	40.90	2.78	88.29	4.50	504.99	4.64	103.94	5.91	869.04	38.01	2025.73	60.85	31.28	1.17	26.49	0.93	798.86	26.85
H. montbretii	mb1	mb	2.00	0.05	18.03	1.08	19.30	1.07	466.51	26.00	261.19	14.17	n. d.	n. d.	354.18	7.79	14.77	0.04	4.12	0.40	51.07	3.37
H. montbretii	mb2	mb	2.96	0.07	33.11	2.40	71.70	6.97	422.40	2.21	127.71	5.79	2121.70	126.13	850.41	13.72	25.21	0.10	51.00	1.16	52.00	4.30
H. richerii subsp. *grisebachii*	rg1	rg	2.71	0.11	22.74	1.63	57.50	0.44	598.68	58.46	163.65	9.81	474.32	4.40	1103.31	28.22	14.01	0.30	177.83	8.51	54.07	5.14
H. richerii subsp. *grisebachii*	rg2	rg	3.03	0.04	19.89	0.45	58.65	0.44	431.91	13.23	215.96	17.52	807.79	4.92	1536.65	18.35	15.02	0.10	4.43	0.35	42.37	1.72
H. richerii subsp. *grisebachii*	rg3	rg	3.11	0.07	30.94	1.25	121.05	10.46	690.15	50.48	192.61	2.00	1223.33	60.37	1553.36	16.22	16.77	0.42	5.35	0.04	56.20	2.75
H. richerii subsp. *grisebachii*	rg4	rg	2.82	0.14	40.14	0.76	84.65	0.39	n. d.	n. d.	130.02	7.62	1096.93	2.00	n. d.	n. d.	27.96	0.31	61.04	1.73	57.85	4.24
H. richerii subsp. *grisebachii*	rg5	rg	3.18	0.13	32.47	2.40	103.08	10.03	n. d.	n. d.	119.58	5.19	975.28	17.34	1397.91	79.65	53.10	0.22	2.12	0.16	156.98	12.64
H. rochelii	ro1	ro	2.88	0.12	15.22	1.26	36.53	0.73	352.33	31.13	238.68	4.38	856.10	31.88	2504.61	99.31	102.31	3.71	20.27	0.31	57.06	1.98
H. rochelii	ro2	ro	5.84	0.10	27.89	1.67	54.84	3.42	307.73	30.11	122.10	0.63	744.33	40.82	6321.77	107.30	17.16	0.57	10.25	0.72	39.81	0.69
H. rochelii	ro3	ro	3.35	0.00	10.91	0.65	33.70	2.58	207.33	14.83	172.15	1.95	1232.23	70.95	1269.89	63.21	23.41	0.62	12.58	0.90	52.86	1.25
H. rochelii	ro4	ro	3.43	0.05	21.08	1.28	45.31	1.17	323.91	22.44	174.52	8.46	481.43	11.57	n. d.	n. d.	17.43	0.04	2.46	0.03	61.03	5.61
H. rochelii	ro5	ro	2.56	0.11	11.71	0.17	40.28	2.28	262.34	17.84	183.39	12.44	196.98	3.09	619.31	31.33	12.13	0.49	5.26	0.23	60.66	2.29
H. rumeliacum	ru1	ru	2.88	0.06	15.59	1.34	25.09	0.92	282.10	9.25	243.48	16.65	689.89	40.50	3649.45	88.46	16.72	0.10	10.00	0.84	56.22	5.38
H. rumeliacum	ru2	ru	3.57	0.05	24.83	1.84	57.23	5.58	506.76	35.23	185.35	4.45	829.06	0.37	1851.06	60.34	27.99	0.88	5.48	0.06	60.78	3.30
H. rumeliacum	ru3	ru	3.47	0.01	39.06	1.17	69.66	2.79	421.41	31.93	171.38	5.35	789.77	33.16	1524.30	57.29	26.04	1.45	2.65	0.16	65.46	6.48
H. rumeliacum	ru4	ru	3.10	0.15	21.05	1.36	37.77	0.10	369.28	16.17	191.95	1.08	1353.23	15.20	n. d.	n. d.	18.03	0.81	6.09	0.11	145.84	3.00
H. rumeliacum	ru5	ru	2.94	0.08	18.60	0.34	33.69	1.81	377.59	29.43	211.00	6.46	1158.42	14.67	2059.61	42.52	18.47	0.20	31.66	0.77	54.49	3.23
H. rumeliacum	ru6	ru	3.98	0.11	17.46	1.14	36.46	0.32	246.13	0.28	232.80	0.19	870.26	49.45	n. d.	n. d.	26.93	0.09	8.65	0.70	77.75	1.64
H. rumeliacum	ru7	ru	3.15	0.09	22.91	1.28	31.88	2.38	294.82	21.37	231.77	13.33	1272.12	47.14	n. d.	n. d.	21.45	0.38	16.59	0.22	86.20	7.46
H. rumeliacum	ru8	ru	4.00	0.13	22.06	0.72	51.22	5.01	515.65	8.14	149.08	5.89	1301.21	15.59	n. d.	n. d.	30.24	0.75	12.95	0.14	65.27	1.36
H. rumeliacum	ru9	ru	3.39	0.10	23.62	2.00	40.42	3.69	277.63	3.65	212.38	15.39	1577.85	15.89	3404.38	197.03	26.26	0.23	9.22	0.04	61.09	0.44
H. rumeliacum	ru10	ru	3.54	0.13	27.42	0.54	31.08	0.80	413.42	33.51	196.28	6.48	1339.93	61.27	1444.87	29.27	19.34	0.79	9.21	0.29	65.39	3.04
H. rumeliacum	ru11	ru	3.00	0.05	23.07	1.76	45.11	4.07	492.61	22.53	197.86	7.53	n. d.	n. d.	1222.44	28.01	76.05	3.33	4.47	0.01	56.74	3.66
H. rumeliacum	ru12	ru	4.09	0.09	28.20	0.13	48.45	1.22	307.03	4.24	193.86	9.77	1511.19	19.90	n. d.	n. d.	16.45	0.74	4.23	0.33	72.22	5.00
H. rumeliacum	ru13	ru	2.42	0.03	19.92	0.76	47.63	1.24	360.77	31.79	272.05	9.07	766.42	21.69	n. d.	n. d.	20.66	0.49	13.69	0.53	52.10	0.89
H. rumeliacum	ru14	ru	3.52	0.08	35.60	2.20	53.29	2.67	519.64	32.69	151.02	5.07	n. d.	n. d.	1278.28	4.63	53.80	3.17	8.87	0.36	49.57	2.54
H. rumeliacum	ru15	ru	3.16	0.11	31.79	0.32	38.16	0.83	462.91	19.51	169.41	10.82	2146.17	125.25	2672.82	63.50	29.11	1.33	5.08	0.08	67.95	0.63
H. rumeliacum	ru16	ru	3.91	0.07	36.70	3.23	60.54	5.77	313.98	12.13	103.10	3.33	1281.19	14.01	1452.75	7.64	48.61	1.49	4.10	0.15	139.18	4.29
H. rumeliacum	ru17	ru	3.11	0.06	25.37	0.35	36.39	1.22	323.69	13.07	227.11	5.86	1610.37	0.94	n. d.	n. d.	21.28	0.54	6.57	0.16	71.58	2.99
H. rumeliacum	ru18	ru	4.46	0.13	41.76	0.21	46.22	1.42	550.58	36.67	157.90	3.56	3870.89	206.99	n. d.	n. d.	33.19	0.55	2.81	0.05	75.57	0.50
H. rumeliacum	ru19	ru	2.45	0.02	21.65	1.93	45.16	2.48	424.58	33.58	169.78	9.32	n. d.	n. d.	3363.36	76.35	19.75	1.09	13.19	0.47	73.41	4.01
H. spruneri	s1	s	3.16	0.03	25.45	0.37	50.74	2.38	479.68	45.34	108.28	7.06	1838.39	66.66	646.77	25.11	27.50	1.31	6.03	0.05	114.07	0.08
H. spruneri	s2	s	2.69	0.02	20.63	1.10	51.52	3.95	263.29	15.95	181.86	3.49	1265.94	2.48	450.67	2.09	15.27	0.85	9.93	0.20	37.15	2.16
