# Supplementary material for: Biological Potential of Hypericum L. Sect. Drosocarpium Species
Source: Life (Basel). 2025 Aug 21;15(8):1332. doi: 10.3390/life15081332 (PMC12387250; doi:10.3390/life15081332)
Supplement: Supplementary file 1 [file life-15-01332-s001.zip › Proof_corrected_Suppl/Table S3_v1.pdf]

Table S3. Herbal samples used in the research.

| Sample | Sample code | Voucher No. | Taxon | Country | Locality | Date | Legator | Voucher specimen identified by: |
|---|---|---|---|---|---|---|---|---|
| b1 | b | 2-0050 | *Hypericum barbatum* Jacq. 1775 | Serbia | Mt. Zlatibor | 14.07.2014. | Nebojša Kladar, Biljana Božin | prof. dr Goran Anačkov |
| b2 | b | 2-0051 | *Hypericum barbatum* Jacq. 1775 | Serbia | Mt. Stolovi | 23.06.2014. | Nebojša Kladar, Biljana Božin | prof. dr Goran Anačkov |
| b3 | b | 2-0052 | *Hypericum barbatum* Jacq. 1775 | Serbia | Mt. Goč | 23.06.2014. | Nebojša Kladar, Biljana Božin | prof. dr Goran Anačkov |
| b4 | b | 2-0053 | *Hypericum barbatum* Jacq. 1775 | North Macedonia | Galichnik | 14.07.2013. | Nebojša Kladar, Biljana Božin | prof. dr Goran Anačkov |
| b5 | b | 2-0054 | *Hypericum barbatum* Jacq. 1775 | North Macedonia | Tonivoda | 17.07.2013. | Nebojša Kladar, Biljana Božin | prof. dr Goran Anačkov |
| b6 | b | 2-0055 | *Hypericum barbatum* Jacq. 1775 | North Macedonia | Mt. Galičica | 19.07.2013. | Nebojša Kladar, Biljana Božin | prof. dr Goran Anačkov |
| b7 | b | 2-0056 | *Hypericum barbatum* Jacq. 1775 | North Macedonia | Stenje-Konjsko | 31.05.2014. | Nebojša Kladar, Biljana Božin | prof. dr Goran Anačkov |
| b8 | b | 2-0057 | *Hypericum barbatum* Jacq. 1775 | North Macedonia | Mariovo | 01.06.2012. | Nebojša Kladar, Biljana Božin | prof. dr Goran Anačkov |
| b9 | b | 2-0058 | *Hypericum barbatum* Jacq. 1775 | North Macedonia | Mt. Kozjak | 01.06.2014. | Nebojša Kladar, Biljana Božin | prof. dr Goran Anačkov |
| b10 | b | 2-0059 | *Hypericum barbatum* Jacq. 1775 | North Macedonia | Baba Mt. | 24.06.2014. | Nebojša Kladar, Biljana Božin | prof. dr Goran Anačkov |
| b11 | b | 2-0060 | *Hypericum barbatum* Jacq. 1775 | Bosnia and Herzegovina | Suvi do - Žepa | 13.07.2014. | Nebojša Kladar, Biljana Božin | prof. dr Goran Anačkov |
| mb1 | mb | 2-0061 | *Hypericum montbretii* Spach 1836 | Serbia | Pčinja valley | 13.06.2013. | Nebojša Kladar, Biljana Božin | prof. dr Goran Anačkov |
| mb2 | mb | 2-0062 | *Hypericum montbretii* Spach 1836 | North Macedonia | Stenje-Konjsko | 01.05.2014. | Nebojša Kladar, Biljana Božin | prof. dr Goran Anačkov |
| rg1 | rg | 2-0063 | *Hypericum richerii* Vill. 1779 subsp. *grisebachii* (Boiss.) Nyman 1878 | North Macedonia | Šar Mountains | 18.07.2013. | Nebojša Kladar, Biljana Božin | prof. dr Goran Anačkov |
| rg2 | rg | 2-0064 | *Hypericum richerii* Vill. 1779 subsp. *grisebachii* (Boiss.) Nyman 1878 | Serbia | Mt. Kopaonik | 25.07.2014. | Nebojša Kladar, Biljana Božin | prof. dr Goran Anačkov |
| rg3 | rg | 2-0065 | *Hypericum richerii* Vill. 1779 subsp. *grisebachii* (Boiss.) Nyman 1878 | Serbia | Mt. Mokra gora | 26.07.2014. | Nebojša Kladar, Biljana Božin | prof. dr Goran Anačkov |
| rg4 | rg | 2-0066 | *Hypericum richerii* Vill. 1779 subsp. *grisebachii* (Boiss.) Nyman 1878 | Montenegro | Mt. Durmitor | 30.07.2014. | Nebojša Kladar, Biljana Božin | prof. dr Goran Anačkov |
| rg5 | rg | 2-0067 | *Hypericum richerii* Vill. 1779 subsp. *grisebachii* (Boiss.) Nyman 1878 | Montenegro | Mt. Durmitor | 29.07.2014. | Nebojša Kladar, Biljana Božin | prof. dr Goran Anačkov |
| ro1 | ro | 2-0068 | *Hypericum rochelii* Griseb. et Schenk 1852 | Serbia | Mt. Suva planina | 22.06.2014. | Nebojša Kladar, Biljana Božin | prof. dr Goran Anačkov |
| ro2 | ro | 2-0069 | *Hypericum rochelii* Griseb. et Schenk 1852 | Serbia | Sićevo gorge | 29.05.2014. | Nebojša Kladar, Biljana Božin | prof. dr Goran Anačkov |
| ro3 | ro | 2-0070 | *Hypericum rochelii* Griseb. et Schenk 1852 | Serbia | Mt. Rtanj | 21.06.2014. | Nebojša Kladar, Biljana Božin | prof. dr Goran Anačkov |
| ro4 | ro | 2-0071 | *Hypericum rochelii* Griseb. et Schenk 1852 | Serbia | Lazar's canyon | 25.05.2014. | Nebojša Kladar, Biljana Božin | prof. dr Goran Anačkov |
| ro5 | ro | 2-0072 | *Hypericum rochelii* Griseb. et Schenk 1852 | Serbia | Sokobanja | 20.05.2014. | Nebojša Kladar, Biljana Božin | prof. dr Goran Anačkov |
| ru1 | ru | 2-0073 | *Hypericum rumeliacum* Boiss. 1849 | Serbia | Pčinja valley | 30.05.2014. | Nebojša Kladar, Biljana Božin | prof. dr Goran Anačkov |
| ru2 | ru | 2-0074 | *Hypericum rumeliacum* Boiss. 1849 | Serbia | Trgovište | 27.05.2014. | Nebojša Kladar, Biljana Božin | prof. dr Goran Anačkov |
| ru3 | ru | 2-0075 | *Hypericum rumeliacum* Boiss. 1849 | Serbia | Bujanovac | 16.06.2013. | Nebojša Kladar, Biljana Božin | prof. dr Goran Anačkov |
| ru4 | ru | 2-0076 | *Hypericum rumeliacum* Boiss. 1849 | Serbia | Jelašnička gorge | 28.05.2014. | Nebojša Kladar, Biljana Božin | prof. dr Goran Anačkov |
| ru5 | ru | 2-0077 | *Hypericum rumeliacum* Boiss. 1849 | Serbia | Sićevo gorge | 29.05.2014. | Nebojša Kladar, Biljana Božin | prof. dr Goran Anačkov |
| ru6 | ru | 2-0078 | *Hypericum rumeliacum* Boiss. 1849 | Serbia | Mt. Rujan | 25.05.2014. | Vladimir Ranđelović | prof. dr Goran Anačkov |
| ru7 | ru | 2-0079 | *Hypericum rumeliacum* Boiss. 1849 | Serbia | Kamenički vis | 28.05.2014. | Nebojša Kladar, Biljana Božin | prof. dr Goran Anačkov |
| ru8 | ru | 2-0080 | *Hypericum rumeliacum* Boiss. 1849 | Serbia | Boljevac - Knjaževac | 27.05.2014. | Nebojša Kladar, Biljana Božin | prof. dr Goran Anačkov |
| ru9 | ru | 2-0081 | *Hypericum rumeliacum* Boiss. 1849 | Serbia | Zlot river gorge | 25.05.2014. | Nebojša Kladar, Biljana Božin | prof. dr Goran Anačkov |
| ru10 | ru | 2-0082 | *Hypericum rumeliacum* Boiss. 1849 | Serbia | Brestovac spa | 25.05.2014. | Nebojša Kladar, Biljana Božin | prof. dr Goran Anačkov |
| ru11 | ru | 2-0083 | *Hypericum rumeliacum* Boiss. 1849 | Serbia | Prolom spa | 07.07.2013. | Nebojša Kladar, Biljana Božin | prof. dr Goran Anačkov |
| ru12 | ru | 2-0084 | *Hypericum rumeliacum* Boiss. 1849 | Serbia | Dimitrovgrad | 27.05.2014. | Nebojša Kladar, Biljana Božin | prof. dr Goran Anačkov |
| ru13 | ru | 2-0085 | *Hypericum rumeliacum* Boiss. 1849 | Greece | Konitsa | 28.05.2014. | Vladimir Ranđelović | prof. dr Goran Anačkov |
| ru14 | ru | 2-0086 | *Hypericum rumeliacum* Boiss. 1849 | North Macedonia | Mt. Galičica | 31.05.2014. | Nebojša Kladar, Biljana Božin | prof. dr Goran Anačkov |
| ru15 | ru | 2-0087 | *Hypericum rumeliacum* Boiss. 1849 | North Macedonia | Stenje-Konjsko | 31.05.2014. | Nebojša Kladar, Biljana Božin | prof. dr Goran Anačkov |
| ru16 | ru | 2-0088 | *Hypericum rumeliacum* Boiss. 1849 | North Macedonia | Pletvar | 01.06.2014. | Nebojša Kladar, Biljana Božin | prof. dr Goran Anačkov |
| ru17 | ru | 2-0089 | *Hypericum rumeliacum* Boiss. 1849 | North Macedonia | Mariovo | 01.06.2014. | Nebojša Kladar, Biljana Božin | prof. dr Goran Anačkov |
| ru18 | ru | 2-0090 | *Hypericum rumeliacum* Boiss. 1849 | North Macedonia | Mt. Kozjak | 01.06.2014. | Nebojša Kladar, Biljana Božin | prof. dr Goran Anačkov |
| ru19 | ru | 2-0091 | *Hypericum rumeliacum* Boiss. 1849 | North Macedonia | Matka canyon | 30.05.2014. | Nebojša Kladar, Biljana Božin | prof. dr Goran Anačkov |
| s1 | s | 2-0092 | *Hypericum spruneri* Boiss. 1849 | Albania | Kukes-Elbasan | 04.06.2014. | Nebojša Kladar, Biljana Božin | prof. dr Goran Anačkov |
| s2 | s | 2-0093 | *Hypericum spruneri* Boiss. 1849 | Albania | Gracen | 04.06.2014. | Nebojša Kladar, Biljana Božin | prof. dr Goran Anačkov |
